# Prophylaxis for renal patients at risk of COVID-19 infection: results from the intranasal niclosamide randomised, double blinded, placebo controlled arm of the PROTECT-V platform trial

**DOI:** 10.1186/s12879-025-10584-4

**Published:** 2025-02-11

**Authors:** Toby J. L. Humphrey, Wendi Qian, Michael Chen-Xu, Francis Dowling, Katrina Gatley, Rakshya Adhikari, Tracey Hensman, Louise Stockley, Abhinav Bassi, Nikita Bathla, Indranil Dasgupta, Davinder P. S. Dosanjh, Mads Jellingsø, Per Sørensen, Morten Lind Jensen, Anne Weibel Callesen, John R. Bradley, Vivekanand Jha, Morten O. A. Sommer, Thomas F. Hiemstra, Rona M. Smith, PROTECT-V consortium, Bassam Alchi, Abdulfattah Alejmi, Neil Basu, Charlotte Bebb, Samira Bell, Anudita Bhargava, Sunil Bhandari, Coralie Bingham, Kate Bramham, Fergus Caskey, Sourabh Chand, Dhruva Chaudhry, Arpita Ray Chaudhury, Sashidhar Chennamsetty, Nihil Chitalia, Paramit Chowdhury, Simon Curran, Simon Davies, Rachel Davison, Michael Delaney, Vishal Dey, Jonathan Dick, Mahmoud Eid, Ragada El-Damanawi, Sarah Fluck, Rouvick Gama, Christopher Goldsmith, Effrossyni Gkrania-Klotsas, Sian Griffin, Richard Hull, Avinash Ignatius, David Jayne, Colin Jones, Manivarma Kamalnathan, Nitin Kolhe, Tanguy Lafont, Mark Lambie, Sarah Lawman, Thomas Ledson, Liz Lightstone, Bethany Lucas, Viyaasan Mahalingasivam, Patrick Mark, Stephen McAdoo, Kieran McCafferty, Jean Patrick, Narayan Prasad, Nicholas Pritchard, Francesco Rainone, Raja Ramachandran, Vinay Rathore, Manisha Sahay, Alan Salama, Sanjiv Saxena, Sapna Shah, Claire Sharpe, Sebastian Spencer, Jo Taylor, Patrick Trotter, Udaya Udayaraj, Shiva Ugni, Josh Wade, Mona Wahba, James Wason, Martin Wilkie, Ian Wilkinson

**Affiliations:** 1 Cambridge University Hospitals NHS Foundation Trust, Cambridge, UK; 2 University of Cambridge, Cambridge, UK; 3 George Institute for Global Health, New Delhi, India; 4 Department of Renal Medicine, Heartlands Hospital, Birmingham, UK; 5 University of Birmingham, Birmingham, UK; 6 UNION Therapeutics A/S, Hellerup, Denmark; 7 School of Public Health, Imperial College, London, UK; 8 Technical University of Denmark, Lyngby, Denmark

**Keywords:** COVID-19, Prophylaxis, Renal, Clinical trial

## Abstract

**Purpose:**

Despite vaccination, many patients remain vulnerable to COVID-19 infection and poorer outcomes, because of underlying health conditions resulting in sub-optimal vaccine responses. This study aims to demonstrate whether intranasal niclosamide confers additional protection against COVID-19 infection above standard preventative measures including vaccination.

**Methods:**

PROTECT-V (PROphylaxis for paTiEnts at risk of COVID-19 infecTion) is a platform trial testing multiple pre-exposure COVID-19 prophylactic agents in vulnerable patients. This paper reports results from the randomised, double blind, placebo controlled intranasal niclosamide arm.

1651 adult patients on dialysis, with a kidney transplant or renal autoimmune conditions on immunosuppression were randomised from 48 sites (37 UK; 11 Indian). Intranasal niclosamide or matched placebo was administered twice daily, for up to nine months. Primary outcome was time to symptomatic COVID-19 infection.

**Results:**

1651 patients were randomised (826 niclosamide;825 placebo) between February 2021 to November 2022. 655(39.7%) were dialysis patients, 622(37.7%) kidney transplant recipients and 374(22.7%) had renal autoimmune disease. 97.5% patients in the UK and 66.4% patients in India with comparable proportions in both treatment groups had received COVID-19 vaccinations. Despite no adverse safety signal, there was a high withdrawal rate (40% niclosamide;23.8% placebo) due to local upper airway irritation leading to a significantly shorter treatment duration in the niclosamide group). Symptomatic COVID-19 infection during study treatment was observed in 103 patients in the niclosamide group and 133 in the placebo group (estimated hazard ratio 1.02(95%CI 0.79–1.32)).

**Conclusion:**

Intranasal niclosamide did not reduce risk of symptomatic COVID-19 infection in this cohort compared to placebo.

**Trial Registration:**

This study is registered with ClinicalTrials.gov: NCT04870333 (submitted 01/03/2021; posted 03/05/2021), EudraCT: 2020–004144-28 and the Clinical Trials Registry of India (CTRI):#CTRI/2022/03/040802.

**Supplementary Information:**

The online version contains supplementary material available at 10.1186/s12879-025-10584-4.

## Introduction

Several patient groups are vulnerable to coronavirus disease 2019 (COVID-19) infection by virtue of demographics, underlying health conditions or as a consequence of treatments for these conditions, and are also at higher risk of adverse outcomes. This includes patients with kidney disease, individuals on dialysis, in receipt of a kidney transplant or receiving immunosuppression for autoimmune diseases (such as vasculitis, systemic lupus erythematosus (SLE) or glomerulonephritis (GN)).[1] Despite the introduction of widespread vaccination, there remains a need for antigen-independent pre-exposure prophylactic agents in vulnerable patient populations. No vaccine is completely effective, new variants of SARS CoV-2 continue to emerge, and many individuals who are immunocompromised, either due to underlying disease or therapy, are known to mount a suboptimal response to vaccination against viruses.[2–6] Evusheld® (tixagevimab/cilgavimab) was shown to be effective at reducing the risk of developing symptomatic COVID-19 by 77% compared to placebo at 6 months in the PROVENT trial but this study included very few patients with kidney disease or those taking immunosuppressive medications.[7] Evusheld® was approved for use and deployed as a pre-exposure prophylactic agent in North America and several European countries earlier in the pandemic, although these approvals have now been revoked due to viral evolution. At present there are no approved agents for pre-exposure prophylaxis over and above vaccination.

The PROTECT-V platform trial aims to determine if adding pre-exposure prophylaxis treatment to standard prevention management of COVID-19 infection, including behavioural measures (ventilation, masking and social distancing) and vaccination, reduces the risk of symptomatic SARS-CoV-2 infection in vulnerable immunocompromised patients. The PROTECT-V platform is designed with the capacity to test multiple pre-exposure prophylaxis agents, and the study protocol is published.[8] Three separate agents have been included within the platform to date: intranasal niclosamide (a re-purposed anti-helminthic agent); inhaled and intranasal ciclesonide (a corticosteroid); and intravenous sotrovimab (a neutralising monoclonal antibody directed against the spike protein of SARS-CoV-2). [9, 10] The high-risk renal patient populations with end stage kidney disease on dialysis, kidney transplant recipients or renal autoimmune disease were included in the first intervention of niclosamide, as at the time of set up, the renal patient population was particularly impacted. A broader range of vulnerable patient populations have been included with the addition of later interventions as the pandemic has evolved.

This paper reports the results from the niclosamide arm of the trial. Niclosamide, a salicylic acid derivative, an affordable and safe anti-helminthic medication on the World Health Organization’s List of Essential Medicines, has potent anti-SARS-CoV-2 activity in vitro. It emerged as the leading candidate for activity against SARS-CoV-2 in two independent library screens of existing approved drugs.[11–13] Modes of action include modulation of the pH gradient across endosomal membranes inhibiting viral escape, as well as enhanced autophagy through inhibition of S-phase kinase-associated protein 2 leading to activity across all tested SARS-CoV-2 variants.[14–16] As oral niclosamide is poorly absorbed from the gut with low bioavailability, UNION therapeutics developed a stable liquid formulation (UNI911) to be administered via a nasal spray pump maximising delivery of drug to the nasal and upper respiratory tract epithelia, the point of viral entry and replication. [17].

The aim of this arm of the study was to assess whether adding intranasal niclosamide, administered twice daily for up to 9 months to the standard prevention measures against SARS CoV-2 infection was superior to matched placebo for the prevention of symptomatic COVID-19 infection in vulnerable patients with kidney disease.

## Methods

### Study design

The niclosamide arm of the PROTECT-V trial is a randomised, placebo-controlled, double-blind trial comparing intranasal niclosamide to matched placebo. It was the first intervention included in the platform. There was little overlap in recruitment to the subsequent intervention, intravenous sotrovimab, included in the platform. The niclosamide arm participants were recruited from 37 hospital sites in the United Kingdom and 11 sites in India.

### Participants

Patients aged at least 18 years with end-stage kidney disease on dialysis; kidney transplant recipients, and patients with a diagnosis of vasculitis (according to Chapel Hill Consensus Conference 2012 definitions), systemic lupus erythematosus (SLE) or glomerulonephritis receiving at least one immunosuppressive medication were eligible. Patients were excluded if they had significant structural nasal disease; had either positive SARS-CoV-2 swab (PCR or lateral flow test) or symptoms highly suggestive of COVID-19 infection at time of enrolment; had known chronic liver disease or hepatic dysfunction as evidenced by ALT or AST > 3 × upper limit of the normal range; an allergy or hypersensitivity to niclosamide or any of the excipients used; or if they were pregnant, trying to conceive, unwilling to use contraception or breastfeeding. Current participants in another interventional prophylactic or vaccine trial against COVID-19 were also excluded (vaccination as part of standard of care was encouraged for all participants).

This trial was overseen by an independent trial steering committee and independent data monitoring committee. The protocol was approved by the UK Medicines and Healthcare Products Regulatory Agency, South Central Berkshire Research Ethics Committee and the Central Drugs Standard Control Organisation, India and the Ethics Committees of all participating sites in India. The study was conducted in accordance with the Declaration of Helsinki and the EU Clinical Trials Directive. All patients provided written informed consent before enrolment.

### Randomisation and masking

Patients were randomly assigned (1:1) to receive either intranasal niclosamide or matched placebo using a stratified block randomisation method. Randomisation was carried out centrally using a web-based randomisation system. The stratification factors at randomisation were disease sub-group (dialysis, transplant, and autoimmune disease), age (≤ 60 vs > 60 years) and participating site.

### Trial treatment

Participants were randomised to either intranasal niclosamide or matched placebo that was taken for a total of 9 months (36 weeks), or until four weeks after confirmed COVID-19 infection, whichever occurred sooner, or until the common trial close out date when the required number of primary endpoint events had been observed. Intranasal niclosamide (UNION Therapeutics (UNI911)) was given as a 1% niclosamide ethanolamine solution via a nasal spray pump twice daily (140 μL of a 1% niclosamide ethanolamine solution, equivalent to 1.4 mg of niclosamide ethanolamine salt per nostril twice daily) and total daily dose of 5.6 mg niclosamide ethanolamine salt (4.7 mg free niclosamide acid). Based on protein binding and drug kinetics, this twice daily dose would lead to 12-fold higher concentrations than the IC90 in the nasal epithelium, which based on the available antiviral efficacy data against SARS-CoV-2 would limit the ability the virus to establish in the nasal cavity and accordingly act as a prophylactic treatment against COVID-19.

### Trial assessments

A baseline screening visit was conducted face-to-face either at a dialysis or clinic visit with baseline blood tests and a COVID-19 test (PCR or lateral flow) to determine eligibility. Follow up was primarily via remote assessment. Participants were contacted by telephone weekly for the first four weeks and again at week six to record any adverse effects or safety concerns, any indication of COVID-19 infection and to help provide regular participant support. Ongoing assessment from week eight onwards was via two-weekly self-reporting of symptoms, indicative of either COVID-19 infection or adverse effects or tolerability issues with IMP, either online or via telephone consultation depending on patient preference. There was one final face-to-face assessment between four-six weeks after the end of treatment for safety monitoring purposes and to allow blood sample collection for the measurement of COVID-19 antibodies.

### Outcomes

The primary outcome measure was confirmed symptomatic COVID-19 infection during trial treatment. The primary outcome measure event was defined as the presence of both:

1. Confirmed SARS-CoV-2 infection (by either PCR or lateral flow test), and
2. One or more symptoms in keeping with COVID-19, including:

- Respiratory (cough ± sputum and shortness of breath)
- Constitutional (pyrexia/chills, myalgia/arthralgia, fatigue, rash, headache, confusion)
- Gastrointestinal (nausea/vomiting, diarrhoea, abdominal pain, loss of appetite).

The time to confirmed symptomatic COVID-19 infection was calculated from the date of starting trial treatment until date of confirmed COVID-19 infection with above symptoms during trial treatment. Participants for whom a symptomatic COVID-19 infection was not observed, were censored at the date of last trial treatment.

Secondary outcomes included:

1. Confirmed SARS-CoV-2 infection including asymptomatic cases during trial treatment
2. Safety and all-cause mortality
3. Severity of COVID-19 disease (assessed by principal investigator 28 days after the date of positive test or until the date of discharge from hospital, whichever occurred later) according to the adapted WHO ordinal scale.

### Statistical analysis

Based on very limited available data at the time of study design, it was estimated that the 6-month rate of confirmed symptomatic COVID-19 infection would be approximately 15% in the placebo group and 10% in the niclosamide treatment group corresponding to a hazard ratio of 0.648. With a 0.045 significance level, for an overall significance level of 0.05 with two interim analyses using a Lan-DeMets error-spending approach and a power of 90%, the maximum total number events required would be 235. If no interim analyses were performed, a total of 230 primary outcome events was required. Approximately, a total of 1500 patients was required in this event driven study design. A common closeout for the recruitment and trial treatment would be implemented once the specified number of primary outcome events was observed. No interim analyses were performed. The assumptions used for sample size estimation were monitored by the independent data monitoring committee (IDMC). No sample size re-estimation was recommended by the IDMC.

The primary efficacy analysis of this trial, was a Cox regression analysis stratified by country and adjusted by age (> 60 v ≤ 60 years), sex, ethnicity, patient population and known high-risk pre-existing conditions (defined by the presence of chronic lung disease, cancer patients currently receiving treatment, chronic liver disease or HIV infection) for the difference in hazard ratio of the rate of developing the symptomatic COVID-19 infection during the trial treatment period between the niclosamide group and the placebo group. Participants who stopped trial treatment early without COVID-19 infection or stopped trial treatment at the common closeout were censored at the date of last trial treatment or date of common closeout, respectively. Interaction analyses were done to assess the difference in the size of treatment effects in subgroups, which were adjusted in the primary efficacy analyses. A Fine and Gray model was applied for the primary outcome measure (corresponding to the supplementary 1 estimand described in the statistical analysis plan (appendix 1)) with competing risk events pre-specified as the trial treatment stopped early due to patient’s intolerance or toxicity assessed by clinicians [18]. The Aalen-Johansen estimator was used to report cumulative incidence estimation under competing risks. Participants who stopped trial treatment early without COVID-19 infection not due to patient’s intolerance or toxicity assessed by clinicians, or stopped trial treatment at the common closeout were censored at the date of last trial treatment or date of common closeout, respectively. A further sensitivity analysis with the time to COVID-19 infection by 9 months (or 36 weeks) or until the date of common closeout was performed. The sensitivity analysis was based on participants registered with the UK Health Security Agency (UKHSA) in the UK and COVID-19 infection status was obtained via UKHSA with survival status obtained via the UK Office of National Statistics (ONS) registry regardless whether a patient had stopped trial treatment early. This sensitivity analysis would assess the impact on possible different dropout rates between the two groups as there would be no difference on follow up in both arms. Similar to the primary efficacy analysis, a Cox regression model analysis was performed on the confirmed SARS-CoV-2 infection including asymptomatic cases during trial treatment. Participants who stopped trial treatment early without COVID-19 infection or stopped trial treatment at the common closeout were censored at the date of last trial treatment or date of common closeout, respectively. Analyses for other secondary outcome measures were mainly descriptive. The efficacy analyses were based on all eligible participants randomised who received at least one dose of trial treatment (modified intention to treat (mITT)).

## Results

**Fig. 1:**
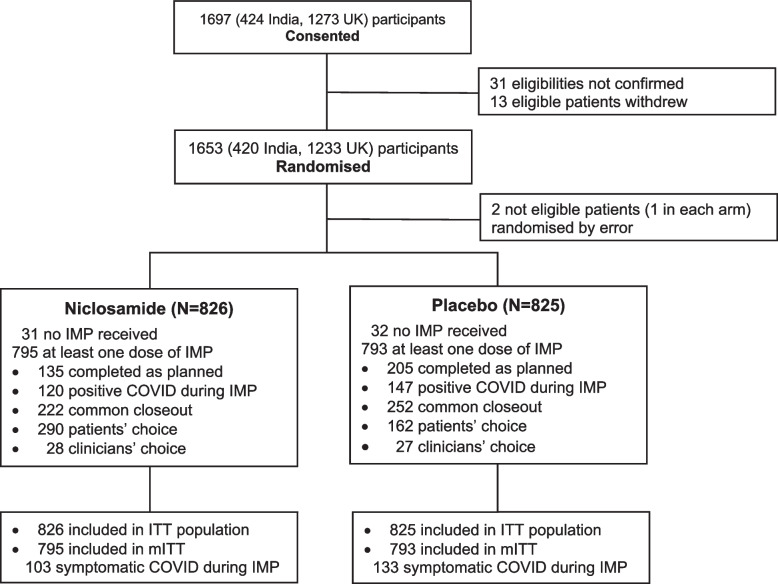
Consort diagram

Between 19 February 2021 and 28 November 2022 1651 patients (1231 UK participants (excluding 2 patients randomised by error, one in each arm), and 420 Indian participants (from March 2022)) from 48 (37 UK, 11 Indian) sites were randomised; 826 to the intranasal niclosamide group and 825 to the placebo group. Of those randomised, 795 patients in the niclosamide group and 793 in the placebo group received at least one dose of trial treatment and are included in the modified ITT (mITT) primary efficacy analysis (Fig. 1). Median age was 55.9 years (range 18.5 – 94.2) and 1049 (63.5%) of participants were male. Individuals recruited in India tended to be younger than those enrolled in the UK (median age 43.2 years (range 18.5–76.2) and 59.5 years (range 19.9–94.2) respectively). The patient populations were as follows: 655 (39.7%) dialysis, 622 (37.7%) renal transplant recipients and 374 (22.7%) had renal autoimmune disease. One hundred and fifty-six (9.4%) patients were considered to be particularly high risk, which was defined as the presence of pre-existing chronic lung disease, chronic liver disease or HIV infection, or cancer patients currently receiving treatment. Overall, 89.6% had received at least one dose of SARS-CoV-2 vaccine at enrolment, although there was a difference between Indian and UK participants with 279/420 (66.4%) patients having received at least one dose of vaccine against SARS-CoV-2 in India compared to 1200/1231 (97.5%) in the UK. However, both groups were well balanced for baseline characteristics (Table 1).

**Table 1:** Baseline characteristics

	Niclosamide		Placebo		Total	
	Number	%	Number	%	Number	%
**Age**						
≤ 60	509	61.6	495	60.0	1004	60.8
> 60	317	38.4	330	40.0	647	39.2
**Sex**						
Female	305	36.9	297	36.0	602	36.5
Male	521	63.1	528	64.0	1049	63.5
**Ethnicity**						
Caucasian	530	64.2	530	64.2	1060	64.2
Black	34	4.1	36	4.4	70	4.2
Asian	249	30.1	247	29.9	496	30.0
Other	13	1.6	12	1.5	25	1.5
**Population**						
Dialysis	334	40.4	321	38.9	655	39.7
Kidney transplant	309	37.4	313	37.9	622	37.7
Vasculitis/GN/ SLE	183	22.2	191	23.2	374	22.7
**Prior infection**	69	8.4	75	9.1	144	8.7
**Prior vaccination**	714	89.9	705	88.9	1419	89.6

*GN* glomerulonephritis, *SLE* systemic lupus erythematosus

Data on 1651 patients. Two of the 1653 randomised found to be ineligible

**Fig. 2:**
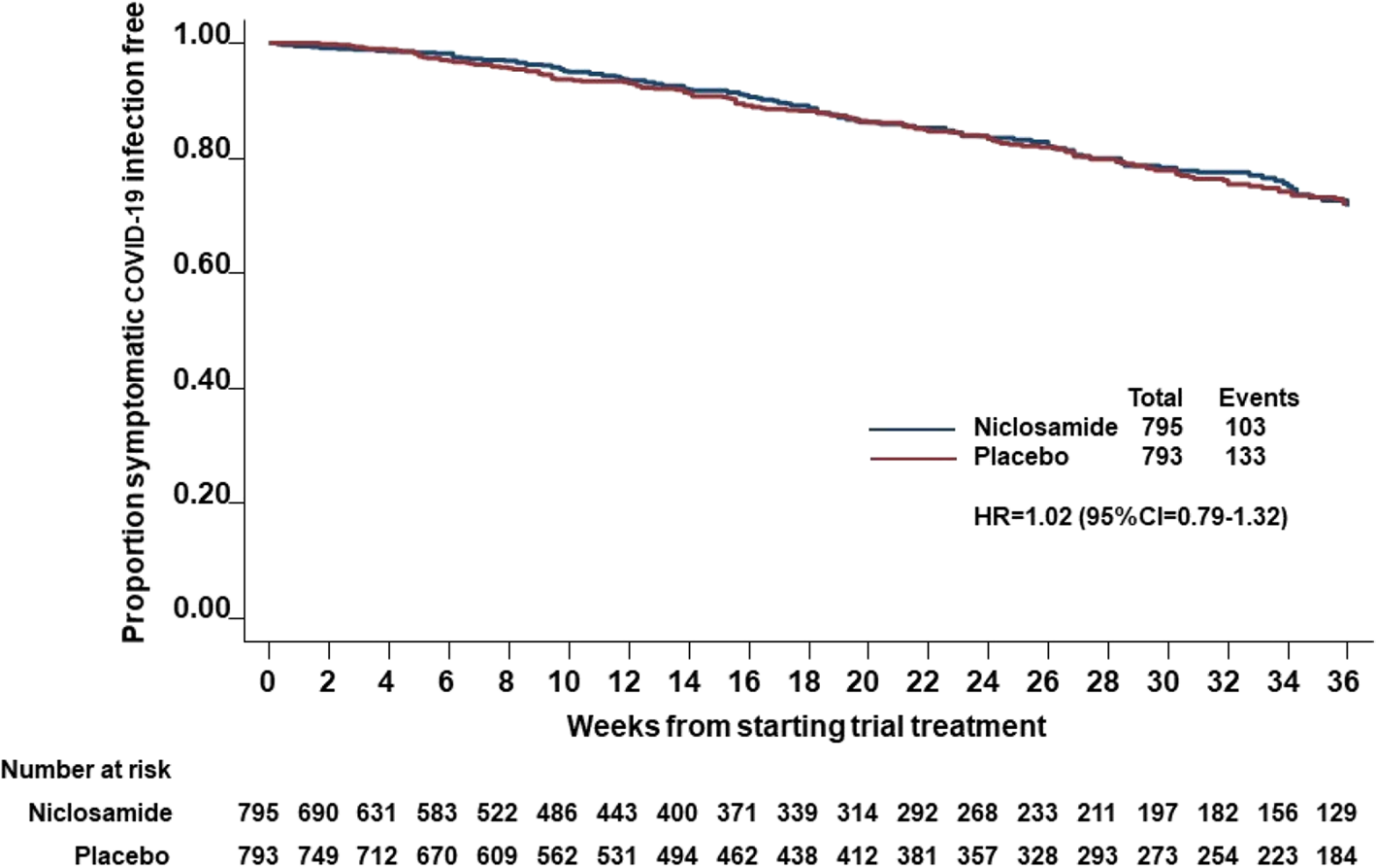
Kaplan–Meier plots on time to confirmed symptomatic COVID-19 infection during trial treatment by treatment allocation

From the 1588 (795 niclosamide and 793 placebo) participants who received at least one dose of allocated study treatment, symptomatic COVID-19 infection was observed in 103 patients in the niclosamide group and 133 patients in the placebo group. With a significantly shorter median follow-up in the niclosamide group (17 weeks) compared to the placebo group (26 weeks) (displayed in supplementary Fig. 1 as number of patients at risk (number of patients who are alive without symptomatic COVID-19 infection) at each time point was smaller in the niclosamide group), the estimated hazard ratio (HR) of niclosamide effect on symptomatic COVID-19 infection was 1.02 (95% CI 0.79–1.32) (Fig. 2). The estimated hazard ratio was stratified by country and adjusted for age, gender, ethnicity, patient population and if particularly high risk. The seemly seemingly unmatched results between the number of primary outcome measure events observed by treatment arm and the estimated hazard ratio are due to the imbalance in dropout between the two groups and can be further explained by the ratio of incidence rate of symptomatic COVID-19 infection per 100 person-years during trial treatment with niclosamide over placebo being 0.95 (95% CI = 0.73. 1.22). The only baseline covariate with a significant influence on development of symptomatic COVID-19 infection was age with a hazard ratio of 0.72 (95% CI 0.54–0.94) in favour of age over 60 years (Supplementary Table 1). There was no evidence that niclosamide treatment effects differed between different pre-specified subgroups of age, sex, ethnicity, patient population (dialysis, transplant or autoimmune renal disease) or high-risk sub-group (supplementary Fig. 2). A total of 267 (120 niclosamide and 147 placebo) patients reported COVID-19 infections including both asymptomatic and symptomatic cases during the trial treatment with a hazard ratio of 1.08 (95% CI 0.85–1.38). Thirty patients were hospitalised with COVID-19 infection (14 in the niclosamide group and 16 in the placebo group). Two deaths due to COVID-19 were reported in each group (Table S2).

Whilst taking trial treatment, patient and clinician reported compliance was good or very good (defined as > 70% planned doses administered) in 82.6% of patients and was comparable between groups (80.9% niclosamide arm and 84.2% placebo). However, withdrawal from trial treatment was common; 318 patients (40%) in the niclosamide group and 189 (23.8%) in the placebo group stopped trial treatment early. Of the 290 patients in the niclosamide group who withdrew due to patient choice, 176 (60.7%) cited local intolerance compared to 61 of 162 (37.7%) patients in the placebo group (Table S3). Median duration of treatment was shorter in the niclosamide group, 102 days (IQR 38–215), than in the placebo group, 153 days (IQR 64–250) (Figure S2). Comparable numbers of patients were withdrawn from each group (28 niclosamide, 27 placebo) due to clinician choice. Clinicians cited toxicity as the reason for withdrawal in 6 patients, 5 in the niclosamide group and 1 in the placebo group. Of these, three patients were reported to have experienced a suspected unexpected serious adverse reaction (SUSAR) (2 cases of epistaxis and one case of thrombocytopaenia). Serious adverse events (SAEs), unrelated to study medication, were common in this patient population. Of the 274 SAEs reported after the trial treatment was commenced, 131 occurred in the niclosamide group and 143 in the placebo group, with 93 and 97 patients in these groups experiencing at least one SAE respectively (Table S4).

**Fig. 3:**
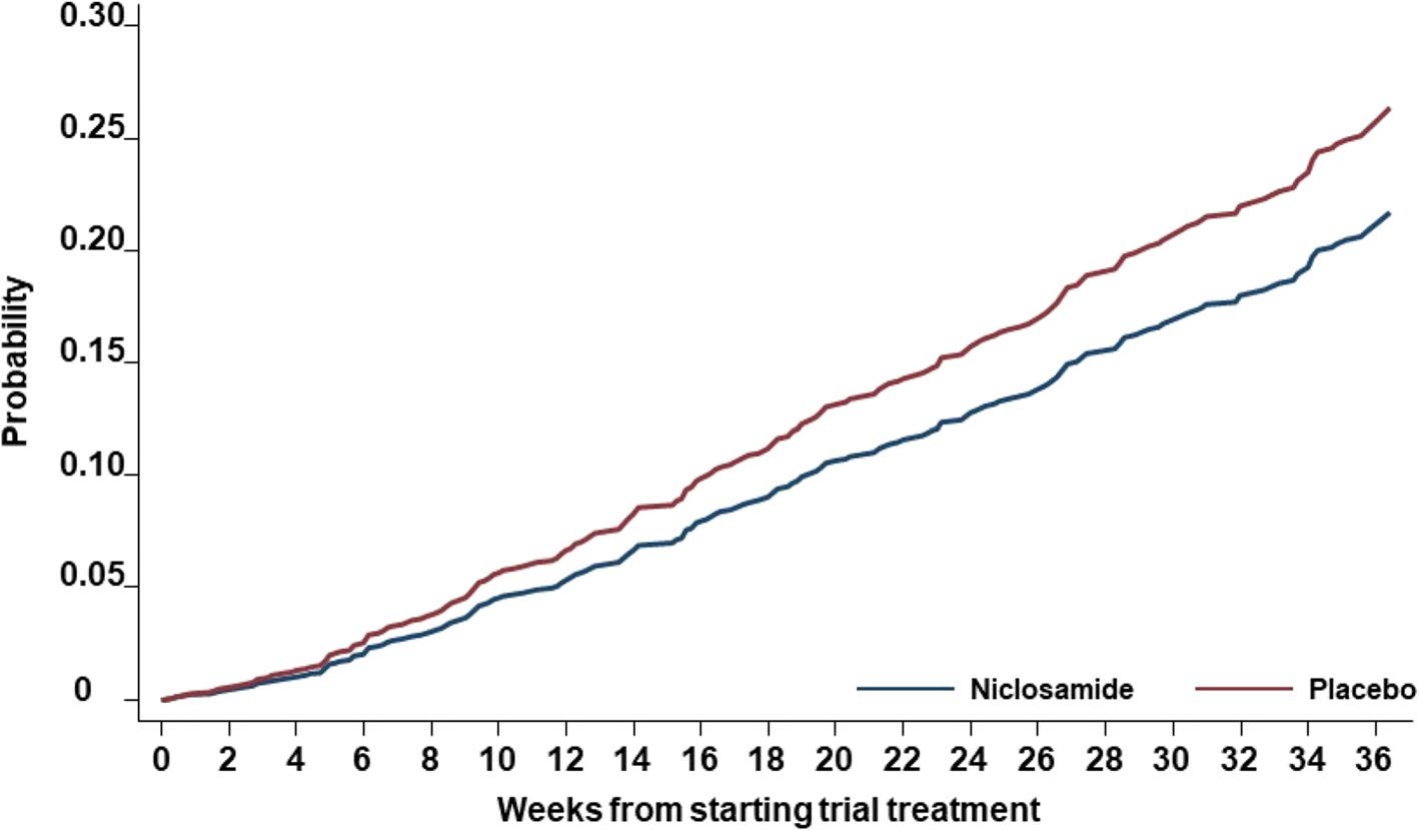
Cumulative incidences of symptomatic COVID-19 infection plots with competing events pre-specified as stopping trial treatment early due to intolerance reported by the patient or toxicity assessed by the treating clinician. Cumulative incidences of symptomatic COVID-19 infection at 12, 24 and 36 weeks were 5.35% (95% CI 4.09–7.01), 12.8% (95% CI 10.6–15.3) and 21.1% (95% CI 17.9–25.0) in the niclosamide group and 6.6% (95% CI 5.3–8.3), 15.7 (95% CI 13.2–18.6) and 25.7% (95% CI 22.4–29.6) in the placebo group

With competing events pre-specified as stopping trial treatment early due to intolerance reported by the patient or toxicity assessed by the treating clinician, the estimated sub-distribution HR on the time to confirmed symptomatic COVID-19 infection using the Fine and Gray model was 0.77 (95% CI 0.599–0.999) for niclosamide group compared to placebo group and the Aalen-Johansen cumulative incidences of symptomatic COVID-19 infection at 12, 24 and 36 weeks were 5.35% (95% CI 4.09–7.01), 12.8% (95% CI 10.6–15.3) and 21.1% (95% CI 17.9–25.0) in the niclosamide group and 6.6% (95% CI 5.3–8.3), 15.7 (95% CI 13.2–18.6) and 25.7% (95% CI 22.4–29.6) in the placebo group respectively (Fig. 3).

**Fig. 4:**
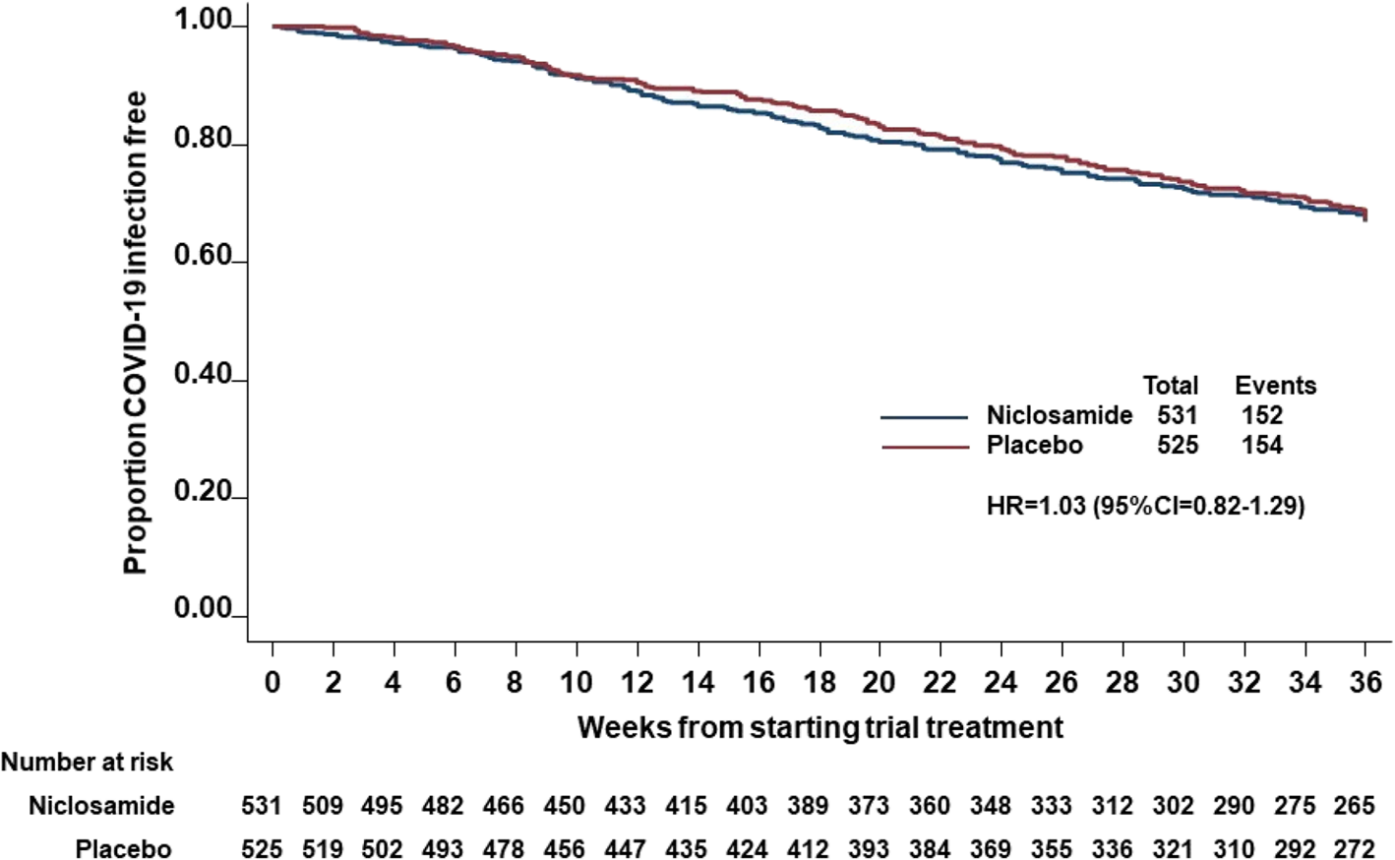
Kaplan–Meier plots on time to COVID-19 infection (symptomatic and asymptomatic) reported using UKHSA data

Following early withdrawal from trial treatment, SARS-CoV-2 results continued to be collected via UKHSA for participants in England for 36 weeks from randomisation or until the common close out date, whichever occurred sooner, for those individuals who provided consent to ongoing data linkage. Data on 1056 patients was available and 306 cases of COVID-19 infection were observed; 152 in the niclosamide group and 154 in the placebo group giving a hazard ratio of 1.03 (95% CI 0.82–1.29) (Fig. 4) and no evidence of difference in follow-up between two groups. In addition, there were no clear evidence of differences in distributions of age, gender, ethnicity, patient population or high risk for completers between two groups or non-completers between two groups.

## Discussion

Intranasal niclosamide did not reduce the risk of symptomatic COVID-19 infection in this vulnerable population of patients with kidney disease compared to placebo. Furthermore, it did not reduce the severity of COVID-19 infections or rates of hospitalisation and no benefit was shown in any pre-defined subgroup analyses. Despite no adverse safety signal, patients receiving intranasal niclosamide had a higher withdrawal rate (40%) compared to placebo (23.8%) due to local nasal and upper airway irritation.

Although the number of symptomatic COVID-19 infections (103 niclosamide, 133 placebo) was 30 more in the placebo group, the total duration of trial treatment was shorter in the niclosamide group (median 7 weeks). There was no difference in the rate of number of symptomatic COVID-19 infections over total duration of trial treatment in each group. When an event of stopping trial treatment early due to intolerance or toxicity (181 niclosimade, 62 placebo) was treated as a competing risk for a confirmed symptomatic COVID-19 infection, the estimated sub-distribution HR was consistent with the number of primary outcome events, and the estimated cumulative incidences of symptomatic COVID-19 infection at 6 months (24 weeks) were 12.8% in the niclosamide group and 15.7% in the placebo group. Considering the high intolerance with niclosamide treatment, this estimated small difference of 2.9% is not clinically meaningful. As a high proportion of patients experienced the competing events (243 (237 intolerance and 6 toxicity)) and they occurred more in the niclosamide arm (181 vs 62), the results considering the competing risk should be interpreted in an exploratory manner.

The differential withdrawal and associated attrition bias is a limitation of this study, despite being a randomised controlled trial. This was not expected during the design stage of the study. Due to pandemic-related constraints and in order to minimise patient risk, the pragmatic decision was made at the study design stage, to cease follow-up if patients discontinued treatment, since the primary hypothesis was efficacy whilst taking treatment. This approach is open to criticism due to the bias it may introduce. However, in order to address this, a pre-planned sensitivity efficacy analysis conducted using routinely collected COVID-19 infection results from UKHSA (formerly Public Health England), out to 9 months for all individuals in England, who consented to ongoing linkage despite withdrawal of therapy (over 1000 patients in total). The medial follow-up on time to any COVID-19 infection (regardless on or after trial treatment) was similar between the two groups. This analysis had a comparable hazard ratio, which provides confidence in the study results. Further limitations include the inability to control for behaviours that may impact on infection risk, such as mask wearing or shielding, and the fact that this guidance changed throughout the course of the study. However, since this was a blinded study, it would be expected that with a large number of participants, the factors would be balanced across groups.

The major strengths of this study are its size, being the largest pre-exposure prophylaxis study of a repurposed agent to be conducted globally, and the high event rate with 236 cases of symptomatic COVID-19 infection being reported. Furthermore, participants were enrolled in both UK and India, reflecting different patient characteristics and risk factors, practice patterns, and health care systems. It also highlights the potential for collaboration between academic, charity and industry partners across international borders and it focuses on patient populations often excluded from clinical trials due to complex underlying health conditions, comorbidity and numerous medications.

Vaccination has dramatically changed the course of the COVID-19 pandemic, but there remain a considerable number of individuals who mount suboptimal vaccine responses either due to their underlying health conditions or therapy received for them, who remain vulnerable to COVID-19 infection and adverse outcomes. The speed of evolution of the SARS-CoV-2 virus poses further challenges to variant-specific vaccines and targeted agents. Consequently, there remains a major need for variant agnostic pre-exposure prophylactic agents for patients who mount suboptimal vaccine responses including patients with renal disease. Nicolsamide was an appealing drug to study, as in addition to it being a leading candidate in drug library screens, by virtue of it’s mechanism of action, the risk of viral evolution and variant development was thought to have minimal impact on it’s potential effect. Although, the selected dose was based on non-clinical PK and Phase 1 human data, it is possible that a protective effect against SARS CoV-2 infection may have be seen with higher doses. However, in view of the poor tolerability due to local irritation of this formulation, this is not a feasible approach.

As the pandemic has evolved so has the PROTECT-V trial platform, and it has now expanded to include many more patients beyond those with renal diseases to include individuals with haematological or oncological diagnoses receiving chemotherapy, other autoimmune diseases receiving immune suppressive therapies, all solid organ and haematopoietic stem cell transplant recipients and those with primary or secondary immunodeficiencies. Sotrovimab 2000 mg intravenously compared to matched placebo is currently under evaluation, and other potential agents for PROTECT-V are being considered.

In conclusion, amongst vulnerable patients with kidney disease at risk of SARS-CoV-2 infection, pre-exposure prophylaxis with intranasal niclosamide spray at this dose did not reduce the risk of COVID-19 infection compared to placebo in this patient population. Despite no adverse safety signal, patients receiving intranasal niclosamide had a higher withdrawal rate compared to placebo in part due to local nasal and upper airway irritation, which complicated the analysis, but also precludes consideration of higher doses.

## Supplementary Information

Supplementary Material 1. Table S1. Effect of baseline covariates on development of symptomatic COVID-19 infection. Table S2: Severity of COVID-19 infection during treatment according to adapted WHO ordinal scale. Table S3. Proportion of patient reporting moderate symptoms, whilst receiving treatment, at any time point from starting treatment. Table S4. Serious adverse events reported during the trial in subjects who received at least one dose of Investigational Medicinal Product (IMP). SOC– System Organ Class; PT – Preferred Term. Figure S1.  Kaplan-Meier plots on time on trial treatment without confirmed symptomatic COVID-19 infection by treatment allocation. Figure S2. Forest plot of time to confirmed symptomatic COVID-19 infection by baseline characteristics. *p* value of the associated interaction with the treatment allocation is presented next to each of the subgroup headings.

## Data Availability

De-identified participant data can be shared on reasonable request to the corresponding author.
