# Supplementary material for: Prophylaxis for renal patients at risk of COVID-19 infection: results from the intranasal niclosamide randomised, double blinded, placebo controlled arm of the PROTECT-V platform trial
Source: BMC Infect Dis. 2025 Feb 11;25:204. doi: 10.1186/s12879-025-10584-4 (PMC11818026; doi:10.1186/s12879-025-10584-4)
Supplement: Supplementary file 1 — Supplementary Material 1. Table S1. Effect of baseline covariates on development of symptomatic COVID-19 infection. Table S2: Severity of COVID-19 infection during treatment according to adapted WHO ordinal scale. Table S3. Proportion of patient reporting moderate symptoms, whilst receiving treatment, at any time point from starting treatment. Table S4. Serious adverse events reported during the trial in subjects who received at least one dose of Investigational Medicinal Product (IMP). SOC– System Organ Class; PT – Preferred Term. Figure S1. Kaplan-Meier plots on time on trial treatment without confirmed symptomatic COVID-19 infection by treatment allocation. Figure S2. Forest plot of time to confirmed symptomatic COVID-19 infection by baseline characteristics. p value of the associated interaction with the treatment allocation is presented next to each of the subgroup headings. [file 12879_2025_10584_MOESM1_ESM.docx]

Supplementary Figure 1: Kaplan-Meier plots on time on trial treatment without confirmed symptomatic COVID-19 infection by treatment allocation


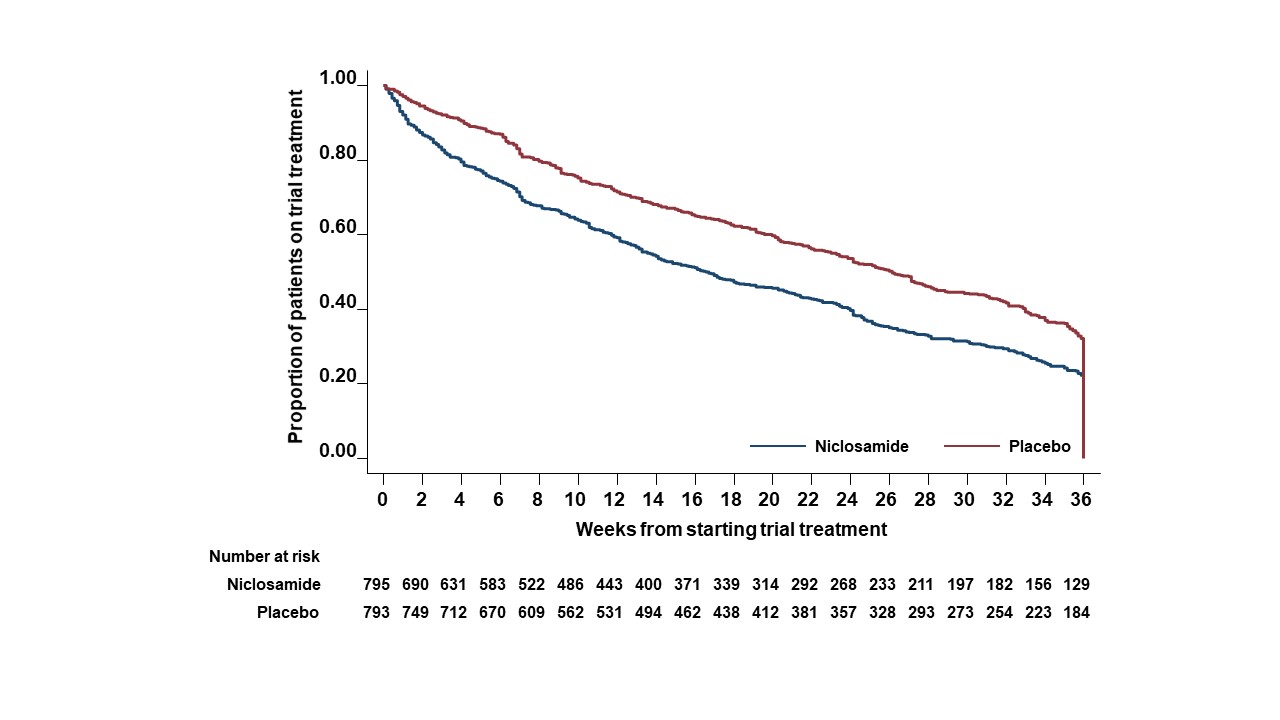


Supplementary Figure 2: Forest plot of time to confirmed symptomatic COVID-19 infection by baseline characteristics


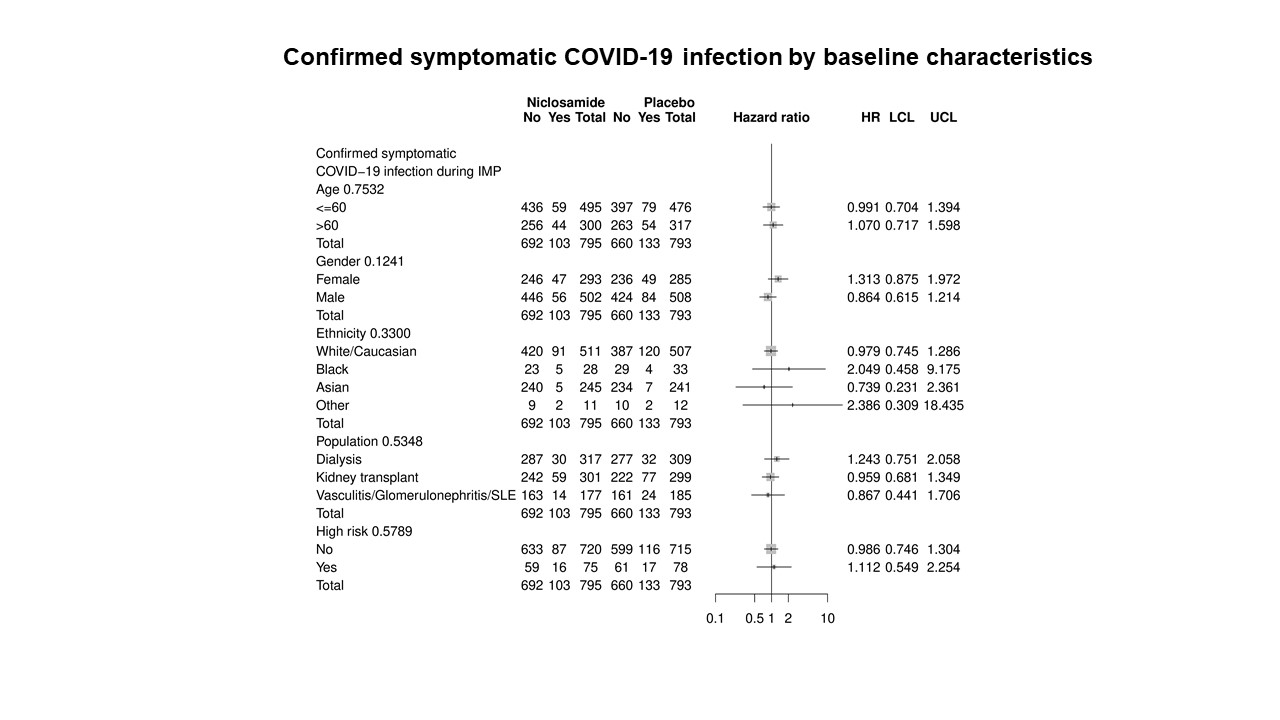


p value of the associated interaction with the treatment allocation is presented next to each of the subgroup headings

Supplementary Table 1: Effect of baseline covariates on development of symptomatic COVID-19 infection

| **Parameter** |  | **Hazard Ratio** | **95% Hazard Ratio Confidence Limits** | |
| --- | --- | --- | --- | --- |
| **Group** | Niclosamide | 1.020 | 0.788 | 1.320 |
| **Age** | >60 | **0.715** | 0.544 | 0.941 |
| **Sex** | Female | 1.285 | 0.989 | 1.671 |
| **Ethnicity** | Black | 1.702 | 0.610 | 4.749 |
|  | Other | 1.602 | 0.450 | 5.699 |
|  | White/Caucasian | 1.905 | 0.848 | 4.278 |
| **Patient Population** | Dialysis | 1.039 | 0.688 | 1.568 |
|  | Kidney transplant | 1.330 | 0.913 | 1.938 |
| **High risk** | No | 0.722 | 0.495 | 1.055 |

Supplementary Table 2: Severity of COVID-19 infection during treatment according to adapted WHO ordinal scale

| **Confirmed COVID-19 infections during IMP (n=267)** | **Niclosamide** | | **Placebo** | | **Total** | |
| --- | --- | --- | --- | --- | --- | --- |
|  | Total | | Total | | Total | |
|  | Number | % | Number | % | Number | % |
| **Healthy carriers - confirmed SARS-CoV2 infection, no symptoms** | 17 | 14.2 | 14 | 9.5 | 31 | 11.6 |
| **Very mind symptoms, no limitations** | 38 | 31.7 | 35 | 23.8 | 73 | 27.3 |
| **Mild, limitations on activities** | 49 | 40.8 | 80 | 54.4 | 129 | 48.3 |
| **Mild, hospitalised, no oxygen requirement** | 5 | 4.2 | 7 | 4.8 | 12 | 4.5 |
| **Moderate, hospitalised, oxygen via mask or nasal cannulae** | 4 | 3.3 | 3 | 2.0 | 7 | 2.6 |
| **Severe, non-invasive ventilation or high low oxygen** | 2 | 1.7 | 1 | 0.7 | 3 | 1.1 |
| **Critical, ventilation and additional organ support(RRT/ECMO)** | . | . | 2 | 1.4 | 2 | 0.7 |
| **Death** | 2 | 1.7 | 2 | 1.4 | 4 | 1.5 |
| **Not available** | . | . | 1 | 0.7 | 1 | 0.4 |
| **Other** | 3 | 2.5 | 2 | 1.4 | 5 | 1.9 |
| **Total** | 120 | 100.0 | 147 | 100.0 | 267 | 100.0 |

RRT – renal replacement therapy; ECMO - Extra Corporeal Membrane Oxygenation

Supplementary Table 3: Proportion of patient reporting moderate symptoms, whilst receiving treatment, at any time point from starting treatment

| **MODERATE SYMPTOM FROM STARTING IMP** | **Niclosamide** | | **Placebo** | | **Total** | |
| --- | --- | --- | --- | --- | --- | --- |
|  |  |  |  |  |  |  |
|  | Number of patients | % of patients | Number of patients | % of patients | Number of patients | % of patients |
| **Moderate/severe pain in nose** | 89 | **11.4** | 27 | **3.5** | 116 | 7.4 |
| **Spontaneous nose bleeds** | 19 | 2.4 | 8 | 1.0 | 27 | 1.7 |
| **Severe itch/burning sensation in nose** | 97 | **12.4** | 23 | **2.9** | 120 | 7.7 |
| **Blocked nose** | 74 | 9.4 | 59 | 7.5 | 133 | 8.5 |
| **Runny nose/blocked or runny nose** | 118 | 15.1 | 68 | 8.7 | 186 | 11.9 |
| **Hoarse voice** | 27 | 3.4 | 26 | 3.3 | 53 | 3.4 |
| **Tingling of tongue/throat** | 57 | **7.3** | 17 | **2.2** | 74 | 4.7 |
| **Loss of the sense of taste/smell** | 27 | 3.4 | 11 | 1.4 | 38 | 2.4 |
| **Sneeze** | 124 | **15.8** | 35 | **4.5** | 159 | 10.2 |
| **Shortness of breath (SOB) when taking or immediately after IMP** | 22 | 2.8 | 17 | 2.2 | 39 | 2.5 |
| **Cough when taking or immediately after IMP** | 50 | **6.4** | 25 | **3.2** | 75 | 4.8 |

IMP – Investigational Medicinal Product

Supplementary table 4: Serious adverse events reported during the trial in subjects who received at least one dose of Investigational Medicinal Product (IMP)

| **MedDRA SOC** | **MedDRA PT** | **Niclosamide** | **Placebo** | **Total** |
| --- | --- | --- | --- | --- |
|  |  | **Number**  **of events** | **Number of events** | **Number of events** |
| **10005329**  **Blood and lymphatic system disorders** | 10002034 Anaemia | 2 | 2 | 4 |
|  | 10040642 Sickle cell anaemia with crisis | 0 | 1 | 1 |
|  | 10043554 Thrombocytopenia | 1 | 0 | 1 |
|  | **TOTAL** | **3** | **3** | **6** |
| **10007541**  **Cardiac disorders** | 10002388 Angina unstable | 0 | 1 | 1 |
|  | 10003658 Atrial fibrillation | 1 | 2 | 3 |
|  | 10003677 Atrioventricular block second degree | 1 | 0 | 1 |
|  | 10007515 Cardiac arrest | 3 | 3 | 6 |
|  | 10007559 Cardiac failure congestive | 0 | 1 | 1 |
|  | 10020823 Hypertensive heart disease | 1 | 0 | 1 |
|  | 10020919 Hypervolaemia | 1 | 4 | 5 |
|  | 10047302 Ventricular tachycardia | 0 | 1 | 1 |
|  | 10063080 Postural orthostatic tachycardia syndrome | 1 | 0 | 1 |
|  | **TOTAL** | **8** | **12** | **20** |
| **10017947**  **Gastrointestinal disorders** | 10000081 Abdominal pain | 1 | 1 | 2 |
|  | 10009887 Colitis | 0 | 1 | 1 |
|  | 10012735 Diarrhoea | 1 | 2 | 3 |
|  | 10013538 Diverticulitis | 0 | 1 | 1 |
|  | 10013836 Duodenal ulcer | 1 | 1 | 2 |
|  | 10016100 Faeces discoloured | 0 | 1 | 1 |
|  | 10017853 Gastritis | 1 | 0 | 1 |
|  | 10018830 Haematemesis | 0 | 1 | 1 |
|  | 10021518 Impaired gastric emptying | 4 | 0 | 3 |
|  | 10022687 Intestinal obstruction | 0 | 1 | 1 |
|  | 10028034 Mouth ulceration | 0 | 1 | 1 |
|  | 10028813 Nausea | 0 | 1 | 1 |
|  | 10033647 Pancreatitis acute | 0 | 1 | 1 |
|  | 10038063 Rectal haemorrhage | 0 | 1 | 1 |
|  | 10045458 Umbilical hernia | 1 | 0 | 1 |
|  | 10047700 Vomiting | 0 | 1 | 1 |
|  | **TOTAL** | **9** | **14** | **23** |
| **10018065**  **General disorders and administration site conditions** | 10008479 Chest pain | 2 | 1 | 3 |
|  | 10011906 Death | 1 | 0 | 1 |
|  | 10025482 Malaise | 1 | 0 | 1 |
|  | 10037660 Pyrexia | 0 | 1 | 1 |
|  | 10064687 Device related infection | 1 | 0 | 1 |
|  | **TOTAL** | **5** | **2** | **7** |
| **10019805 Hepatobiliary disorders** | 10008629 Cholelithiasis | 1 | 0 | 1 |
|  | 10023126 Jaundice | 0 | 1 | 1 |
|  | **TOTAL** | **1** | **1** | **2** |
| **10021428**  **Immune system disorders** | 10021263 IgA nephropathy | 1 | 0 | 1 |
|  | 10023439 Kidney transplant rejection | 1 | 0 | 1 |
|  | 10042945 Systemic lupus erythematosus | 0 | 1 | 1 |
|  | 10044439 Transplant rejection | 0 | 1 | 1 |
|  | 10049169 Pancreas transplant rejection | 1 | 0 | 1 |
|  | 10061626 Allergy to chemicals | 1 | 0 | 1 |
|  | **TOTAL** | **4** | **2** | **6** |
| **10021881**  **Infection and infestations** | 10003997 Bacteraemia | 1 | 0 | 1 |
|  | 10014665 Endocarditis | 1 | 0 | 1 |
|  | 10017888 Gastroenteritis | 2 | 1 | 3 |
|  | 10017918 Gastroenteritis viral | 1 | 0 | 1 |
|  | 10021784 Infected skin ulcer | 1 | 4 | 5 |
|  | 10022000 Influenza | 0 | 1 | 1 |
|  | 10024968 Lower respiratory tract infection | 6 | 2 | 8 |
|  | 10028885 Necrotising fasciitis | 1 | 0 | 1 |
|  | 10034674 Peritonitis | 0 | 1 | 1 |
|  | 10035664 Pneumonia | 3 | 4 | 7 |
|  | 10037596 Pyelonephritis | 3 | 1 | 4 |
|  | 10040047 Sepsis | 3 | 2 | 5 |
|  | 10040872 Skin infection | 0 | 1 | 1 |
|  | 10046571 Urinary tract infection | 3 | 3 | 6 |
|  | 10048709 Urosepsis | 5 | 2 | 7 |
|  | 10048762 Tooth infection | 0 | 1 | 1 |
|  | 10053840 Bacterial sepsis | 0 | 1 | 1 |
|  | 10054236 Clostridium difficle infection | 2 | 0 | 2 |
|  | 10057847 Biliary sepsis | 0 | 1 | 1 |
|  | 10062352 Respiratory tract infection | 0 | 1 | 1 |
|  | 10064687 Device related infection | 0 | 1 | 1 |
|  | 10069767 H1N1 influenza | 0 | 1 | 1 |
|  | 10069802 Device related | 0 | 1 | 1 |
|  | 10069802 Device related sepsis | 1 | 0 | 1 |
|  | 10073755 Pneumocystis jirovecii pneumonia | 0 | 1 | 1 |
|  | 10075611 Varicella zoster virus infection | 1 | 0 | 1 |
|  | 10080714 Vascular device infection | 0 | 2 | 2 |
|  | 10084268 COVID-19 | 14 | 13 | 27 |
|  | 10084380 COVID 19 Pneumonia | 0 | 3 | 3 |
|  | **TOTAL** | **48** | **48** | **96** |
| **10022117**  **Injury, poisoning and procedural complication** | 10002544 Ankle fracture | 0 | 1 | 1 |
|  | 10003192 Arteriovenous fistula thrombosis | 3 | 0 | 3 |
|  | 10016173 Fall | 1 | 2 | 3 |
|  | 10016454 Femur fracture | 0 | 1 | 1 |
|  | 10041649 Splenic injury | 0 | 2 | 2 |
|  | 10042361 Subdural haematoma | 1 | 0 | 1 |
|  | 10048031 Wound dehiscence | 0 | 1 | 1 |
|  | 10049946 Cervical vertebral fracture | 1 | 0 | 1 |
|  | 10053182 Arteriovenous graft thrombosis | 1 | 0 | 1 |
|  | 10055123 Arteriovenous fistula site haemorrhage | 3 | 0 | 3 |
|  | 10055147 Arteriovenous graft site stenosis | 0 | 1 | 1 |
|  | 10057669 Colon Injury | 0 | 1 | 1 |
|  | 10061161 Pelvic fracture | 1 | 0 | 1 |
|  | 10061394 Upper limb fracture | 1 | 0 | 1 |
|  | 10070476 Haemodialysis complication | 0 | 1 | 1 |
|  | 10074860 Transplant dysfunction | 0 | 1 | 1 |
|  | 10077832 Vascular access malfunction | 3 | 2 | 5 |
|  | 10083433 Foreign body in throat | 1 | 0 | 1 |
|  | **TOTAL** | **16** | **13** | **29** |
| **10022891 Investigations** | 10004782 Biopsy kidney | 0 | 1 | 1 |
|  | 10024690 Liver function test abnormal | 0 | 1 | 1 |
|  | 10060795 Hepatic enzyme increased | 0 | 1 | 1 |
|  | **TOTAL** | **0** | **3** | **3** |
| **10027433**  **Metabolism and nutrition disorders** | 10012671 Diabetic ketoacidosis | 1 | 0 | 1 |
|  | 10016803 Fluid overload | 2 | 0 | 2 |
|  | 10020583 Hypercalcaemia | 1 | 0 | 1 |
|  | 10020646 Hyperkalaemia | 3 | 2 | 5 |
|  | 10082630 New onset diabetes after transplantation | 1 | 0 | 1 |
|  | **TOTAL** | **8** | **2** | **10** |
| **10028395 Musculoskeletal and connective tissue disorders** | 10003988 Back pain | 0 | 1 | 1 |
|  | 10023232 Joint swelling | 1 | 0 | 1 |
|  | 10031161 Osteoarthritis | 0 | 1 | 1 |
|  | 10042868 Synovitis | 1 | 0 | 1 |
|  | 10049947 Lumbar vertebral fracture | 1 | 0 | 1 |
|  | 10061224 Limb discomfort | 1 | 0 | 1 |
|  | 10061521 Intervertebral disc disorder | 0 | 1 | 1 |
|  | **TOTAL** | **4** | **3** | **7** |
| **10029104**  **Neoplasms benign, malignant and unspecified** | 10025650 Malignant melanoma | 0 | 1 | 1 |
|  | 10026532 Malignant neoplasm of thorax | 1 | 0 | 1 |
|  | 10027457 Metastases to liver | 0 | 1 | 1 |
|  | 10033609 Pancreatic carcinoma | 1 | 0 | 1 |
|  | 10043966 Tongue neoplasm malignant stage unspecified | 0 | 1 | 1 |
|  | 10057529 Ovarian cancer metastatic | 0 | 1 | 1 |
|  | **TOTAL** | **2** | **4** | **6** |
| **10029205**  **Nervous system disorders** | 10008190 Cerebrovascular accident | 1 | 0 | 1 |
|  | 10019211 Headache | 1 | 0 | 1 |
|  | 10036653 Presyncope | 0 | 1 | 1 |
|  | 10039906 Seizure | 2 | 0 | 2 |
|  | 10041466 Speech disorder | 0 | 1 | 1 |
|  | 10044390 Transient ischaemic attack | 0 | 1 | 1 |
|  | 10061256 Ischaemic stroke | 1 | 1 | 2 |
|  | **TOTAL** | **5** | **4** | **9** |
| **10037175**  **Psychiatric disorders** | 10010305 Confusional state | 0 | 1 | 1 |
|  | 10042464 Suicide attempt | 1 | 0 | 1 |
|  | **TOTAL** | **1** | **1** | **2** |
| **10038359**  **Renal and urinary disorders** | 10002847 Anuria | 1 | 0 | 1 |
|  | 10046555 Urinary retention | 0 | 1 | 1 |
|  | 10046571 Urinary tract infection | 0 | 3 | 3 |
|  | 10062237 Renal impairment | 0 | 2 | 2 |
|  | 10069339 Acute kidney injury | 3 | 0 | 1 |
|  | 10077989 Ureterolithiasis | 0 | 1 | 1 |
|  | **TOTAL** | **4** | **7** | **11** |
| **10038604 Reproductive system and breast disorders** | 10034336 Penis disorder | 0 | 1 | 1 |
|  | TOTAL | 0 | 1 | 1 |
| **10038738**  **Respiratory thoracic and mediastinal disorders** | 10010952 COPD | 0 | 1 | 1 |
|  | 10011224 Cough | 0 | 1 | 1 |
|  | 10013968 Dyspnoea | 1 | 1 | 2 |
|  | 10015090 Epistaxis | 2 | 1 | 3 |
|  | 10021143 Hypoxia | 0 | 1 | 1 |
|  | 10038695 Respiratory failure | 1 | 1 | 2 |
|  | **TOTAL** | **4** | **5** | **9** |
| **10042613**  **Surgical and medical procedures** | 10003190 Arteriovenous fistula operation | 0 | 1 | 1 |
|  | 10020096 Hip arthroplasty | 0 | 1 | 1 |
|  | 10023469 Knee arthroplasty | 1 | 0 | 1 |
|  | 10038533 Renal transplant | 1 | 4 | 5 |
|  | 10052278 Renal and pancreas transplant | 1 | 0 | 1 |
|  | 10057681 Joint arthroplasty | 1 | 0 | 1 |
|  | 10059015 Dialysis device insertion | 0 | 1 | 1 |
|  | 10061916 Prostatectomy | 1 | 0 | 1 |
|  | 10077814 Vascular access placement | 0 | 1 | 1 |
|  | 10086417 Bowel obstruction surgery | 1 | 0 | 1 |
|  | **TOTAL** | **6** | **8** | **14** |
| **10047065**  **Vascular disorders** | 10000358 Accelerated hypertension | 1 | 0 | 1 |
|  | 10009192 Circulatory collapse | 0 | 1 | 1 |
|  | 10017788 Gastric Haemorrhage | 0 | 1 | 1 |
|  | 10021097 Hypotension | 0 | 1 | 1 |
|  | 10031127 Orthostatic hypotension | 0 | 1 | 1 |
|  | 10037377 Pulmonary embolism | 0 | 2 | 2 |
|  | 10042316 Subarachnoid haemorrhage | 0 | 1 | 1 |
|  | 10042361 Subdural haematoma | 1 | 0 | 1 |
|  | 10051055 Deep vein thrombosis | 0 | 1 | 1 |
|  | 10058179 Hypertensive emergency | 1 | 0 | 1 |
|  | 10061255 Ischaemia | 1 | 0 | 1 |
|  | **TOTAL** | **4** | **8** | **12** |

SOC – System Organ Class; PT – Preferred Term
